# The effects of fertility and synchronization variation on seed production in two Chinese fir clonal seed orchards

**DOI:** 10.1038/s41598-022-27151-5

**Published:** 2023-01-12

**Authors:** Fang Wang, Shuaiying Zhang, Peng Zhu, Lianghua Chen, Yuanwei Zhu, Changdong Yang, Rendong Liu, Fengqing Li, Xiong Huang, Hanbo Yang

**Affiliations:** 1 Sichuan Provincial Key Laboratory of Ecological Forestry Engineering on the Upper Reaches of the Yangtze River and National Forestry and Grassland Administration Key Laboratory of Forest Resources Conservation and Ecological Safety on the Upper Reaches of the Yangtze River and Rainy Area of West China Plantation Ecosystem Permanent Scientific Research Base, Institute of Ecology and Forestry, Sichuan Agricultural University, Chengdu, 611130 Sichuan China; 2 Hongya State-Owned Forest Farm and Rainy Area of West China Plantation Ecosystem Permanent Scientific Research Base, Meishan, 620364 Sichuan China; 3 Experimental Center of Subtropical Forestry, Chinese Academy of Forestry, Fenyi, 336600 Jiangxi China

**Keywords:** Plant breeding, Agricultural genetics

## Abstract

Variations in fertility and synchronization information is fundamental to seed orchard management. Our objective was to determine clonal variation and stability in strobili production, phenology, synchronization, and seed production in two generation clonal seed orchards (CSO) of Chinese fir. The number of female and male strobili and the phenology of 42 clones in both the 2.0- and 2.5-generation clonal seed orchards were investigated and recorded to calculate the variation and stability of fertility and synchronization. In both seed orchards, an obvious variation in gamete contribution was found among clones, indicating deviation from random mating. Female receptivity was in the pollen shedding stage, which is favorable to pollination. However, low synchronization (mean *POij* = 0.283) between clones indicated low overlap between female receptivity and pollen shedding. A higher *POij* value within clones than within outcrossing combinations indicated a high risk of selfing in two seed orchards, particularly for early- and late-flowering clones. The number of female strobili and *POij* (as female) significantly influence seed production. Overall, fertility and synchronization variation had notable consequences for seed production. Scientific genetic management is indispensable for promoting fertility uniformity and synchronization to obtain maximal genetic gain.

## Introduction

Seed orchards are an important seed source and are essential for global seed production programs^1^. The genetic gain and gene diversity of seeds from seed orchards are expected to be high as a result of continuing improvements to successive generations^2,3^. For the progeny of seed orchards to reach the highest possible genetic gain, complete synchronization is necessary^4^. However, seed orchards often deviate from their “ideal” expectations^5^. For example, fertility variation has been reported in many tree species^6–8^. The difference in female and male strobili production among clones would cause an imbalance in gamete contributions. Commonly, the imbalance of parental contribution will decrease the seed yield in seed orchard^1^. For instance, a significant difference in female and male strobili caused an unequal contribution to the gamete gene pool in *Cedrus libani* seed stands^1^. The deviation of the effective population number from an ideal population was also reported in *Picea sitchensis*, which indicated the presence of a disproportional contribution by the clones^5^. In a *Pinus sylvestris* seed orchard, a differentiation in gene frequencies was exhibited between the parent and the progeny, which mainly resulted from the variation in the imbalance of female and male gamete contributions^6^. Therefore, knowledge of fertility variation is becoming important to evaluate the maintenance of gene diversity and scientific management in seed orchards.

Studies on phenology and synchronization have contributed to evaluating the level of effective out-crossing and the condition of genetic diversity in seed orchard seed lots^2^. Phenology affects the frequency of gene exchange among clones and the genetic composition of seeds derived from seed orchards^8–10^. Previous studies have documented that phenological variation among clones reduces the effective population size, promotes selfing, and increases the risk of contamination^11^. Data from *P. patula* seed orchards suggest that the overlap between female receptivity in orchards and pollen shedding in natural stands produces a risk of pollen contamination^12^. Conversely, the low synchronization between natural stands and seed orchards of *P. patula* lowers the risk of pollen contamination and high seed quality failure^9^. The pronounced variation of gametic contribution and synchronization in *P. sylvestris* seed orchards causes a substantial drift of gene frequency between the seed orchard and its offspring^6^. Overall, knowledge of phenology and synchronization is fundamental for making scientific management decisions regarding seed orchard activities and advanced seed orchard design, such as supplemental mass pollination (SMP) and parent configuration, to attain a high level of genetic gain^10,12^.

A complete and stable synchronization and balance of gamete contamination are required to reach the intended genetic gains and maximize the genotypic diversity of the progeny in seed orchards^4^. Information about fertility and synchronization is important for seed orchard managers, especially with respect to seed production output. Over a six year period, an obvious difference in phenology existed in a Chinese fir seed orchard, but the flowering order of clones remained stable^13^. Monitoring data in a Korean pine seed orchard for five consecutive years showed that the fertility variation would impact the genetic diversity of seed crops and possible management options^14^. In some previous reports, the stability of fertility, phenological traits, and cone production in some tree species remained stable, and with little change from year to year, so we could make an early evaluation and selection of these traits^9,12,15,16^. An inverse report showed unstable cone production over time in a 1.5-generation seed orchard of *P. koraiensis*, indicating that continuous monitoring was necessary for making wise management proposals^17^. Therefore, knowledge of the stability or variation in fertility and phenology is important for seed orchard management.

Chinese fir (*Cunninghamia laceolata* (Lamb.) Hook.) is an important timber tree species for afforestation in southern China due to its fast growth rate and high wood quality^18,19^. As the first batch of tree species for which seed orchards were established in China, the Chinese fir provided a large amounts of high-quality seeds for artificial restoration. However, unfavorable conditions, such as low seed production and decreased genetic diversity, also occurred in some seed orchards. Little information exists on fertility and phenology, which limits formulations for seed orchard management. Therefore, the objectives of the current study are to evaluate the variation and stability of fertility and synchronization of Chinese fir seed orchards and their effects on seed production.

## Results

### Fertility variation

**Figure 1:**
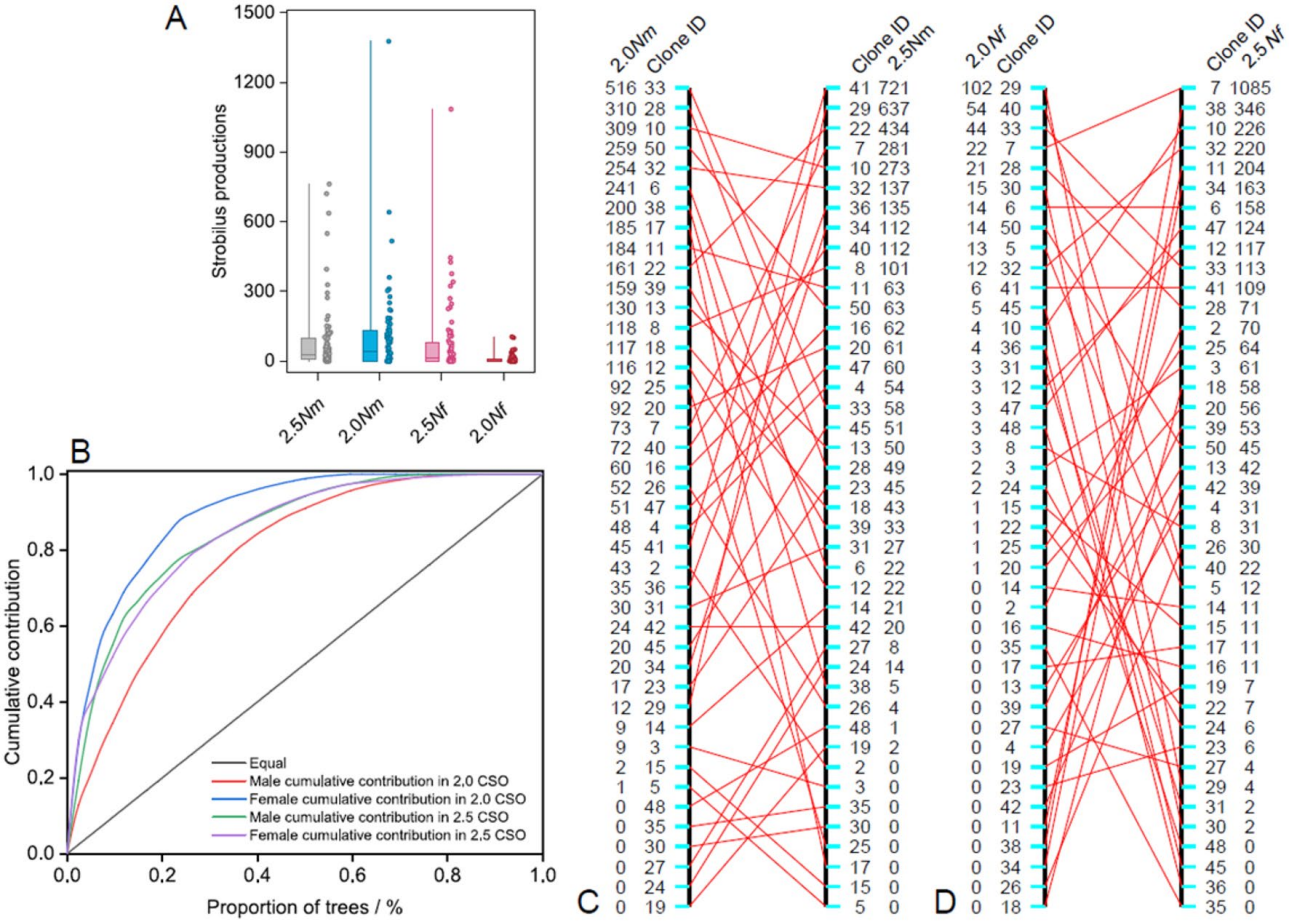
Fertility variation of strobili production in two generations seed orchards of Chinese fir. (**A**) The female and male strobili production for two seed orchards, (**B**) the parental-balance of female and male strobili production in the seed orchards for CSO2 and CSO2.5, (**C**,**D**) the rank order for 42 clones based on average female and male strobili production in two seed orchards. 2.0*Nm* and 2.0*Nf* show the female and male strobili production in CSO2, respectively. Correspondingly, 2.5*Nm* and 2.5*Nf* refer to the female and male strobili production in CSO2.5, respectively.

The numbers of female (*Nf*) and male (*Nm*) strobili varied between the two seed orchards and among clones within a clonal seed orchard (CSO) (Fig. 1A). The *Nf* in the 2.5-generation CSO (CSO2.5) was significantly higher than that in the 2-generation CSO (CSO2) (*F* = 14.63, *P* ˂ 0.001). The analysis of variance (ANOVA) of *Nf* and *Nm* both showed significant differences among clones (*P* ˂ 0.01), but only *Nf* showed a significant difference between the two seed orchards (Table 1). The clonal heritability (*H*_*c*_^2^) was low for *Nf* and *Nm*. The parental balance curves for female and male strobili production significantly deviated from the ideal situation in CSO2 and CSO2.5 (Fig. 1B). The uniformity index of parental gamete contribution (*U*) was 0.428 and 0.291 for males and 0.204 and 0.304 for females in CSO2 and CSO2.5, respectively, which indicated an unbalanced gamete contribution in seed orchards. In CSO2.5, the four clones with the highest contribution (9.5% of total) produced more than 50% strobili. A change in female and male strobili production rank between seed orchards indicated that clone reproductive capacity differed between CSO2 and CSO2.5 (Fig. 1B–D). The female and male strobili productions showed that a very low proportion of clones (i.e., 4.8% for females, 2.4% for males) maintained the same rank in both generation seed orchards. The most abundant seven (16.7% of total) and three (7.1% of total) clones produced more than 50% female and male strobili in CSO2, respectively. Fertility variations of female (*ψf*) and male (*ψm*) parents varied between the two seed orchards, especially for *ψm*, which showed a large difference in fertility between males (Table 2). The sibling coefficient of combined fertility variation (*ψ*) in CSO2.5 was higher than that in CSO2. This was inversely mirrored by the effective number of males (*Npm*) and parents (*Np*) and the relative effective number of females (*Nrf*), males (*Nrm*), and parents (*Nr*).

**Table 1:** Analysis of variance for female (*Nf*) and male (*Nm*) strobili production in seed orchards of Chinese fir.

Source of variation	Variable	*df*	Sum square	Mean square	*F*	*H*_*c*_^2^
Generations	*Nf*	1	383,209	383,209	75.13**	0.099
	*Nm*	1	356	356	0.01	0.487
Clones	*Nf*	41	1,930,480	47,085	9.23**	
	*Nm*	41	2,423,969	59,121	2.16**	
G × C interaction	*Nf*	41	1,899,311	46,325	9.08**	
	*Nm*	41	2,538,429	61,913	2.26**	
Total	*Nf*	83	4,213,000			
	*Nm*	83	4,962,754			

** indicates significant difference at the level of 0.01, the same as below.

**Table 2:** Female and male fertility variation, and the effective number in two seed orchards.

	*ψf*	*ψm*	*Ψ*	*Npf*	*Npm*	*Np*	*Nrf*	*Nrm*	*Nr*
CSO2	5.94	2.35	2.57	7.08	17.89	16.34	0.17	0.43	0.39
CSO2.5	5.13	4.13	3.84	8.19	10.17	10.95	0.20	0.24	0.26

*ψf* variations of female among clones, *ψm* variations of male among clones, *ψ* sibling coefficient of combined fertility variation, *Npf* the effective number of female, *Npm* the effective number of male, *Np* the effective number of parents, *Nrf* the relative effective number of female, *Nrm* the relative effective number of male, *Nr* the relative effective number of parents.

### Flowering phenology

**Figure 2:**
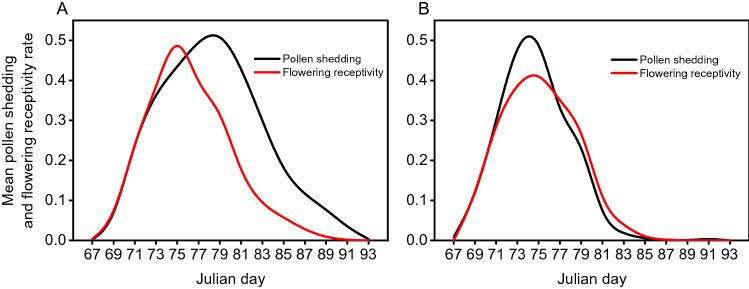
Overall phenology showing the mean pollen shedding rate and female receptivity rate among all clones across the reproductive period for CSO2.5 (**A**) and CSO2 (**B**).

**Figure 3:**
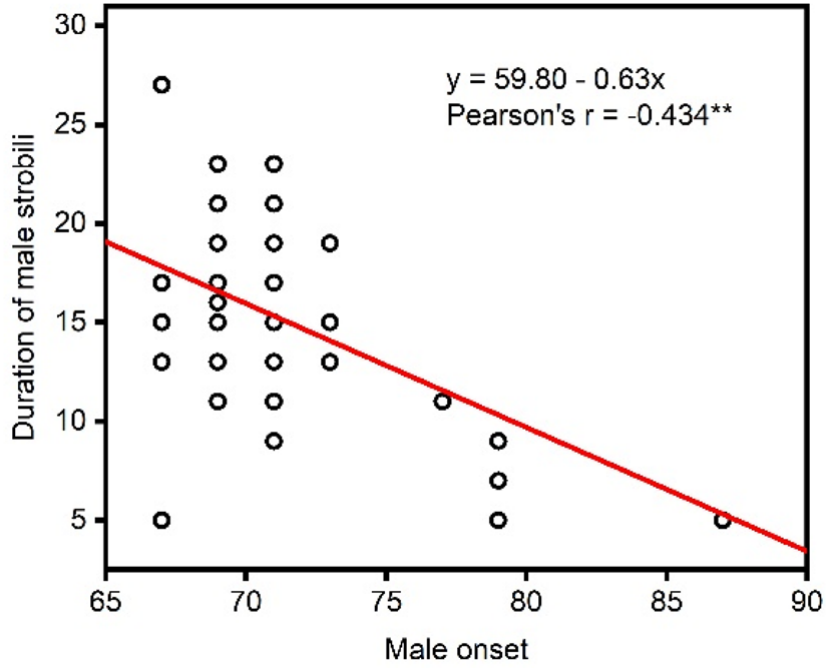
Date of male onset versus duration of pollen shed in seed orchards. **Indicates significant difference at the level of 0.01.

The mean pollen shedding occurred on Julian days 69 and 72 in CSO2 and CSO2.5, respectively, and on average, pollen shedding of the earliest clones started on Julian day 67 in both CSOs (Fig. 2). Variation among clones resulted in a spread of 5–19 and 5–27 days of pollen shedding. The mean female strobili receptivity occurred on Julian days 69 in CSO2 and 70 in CSO2.5, respectively. The stage of female receptivity was under pollen shedding in two seed orchards, which was favorable to pollination. An ANOVA between two seed orchards showed that age had no influence on the clones’ dichogamy (*F* = 1.04, *P* = 0.319). A regression between male onset date and duration of pollen shedding showed that early flowering clones had a longer period of pollen shedding, such as clone 42 in CSO2 (Julian day 67 onset with 13 days duration) and clone 48 in CSO2.5 (Julian day 67 onset with 27 days duration) (Fig. 3).

### Synchronization

**Figure 4:**
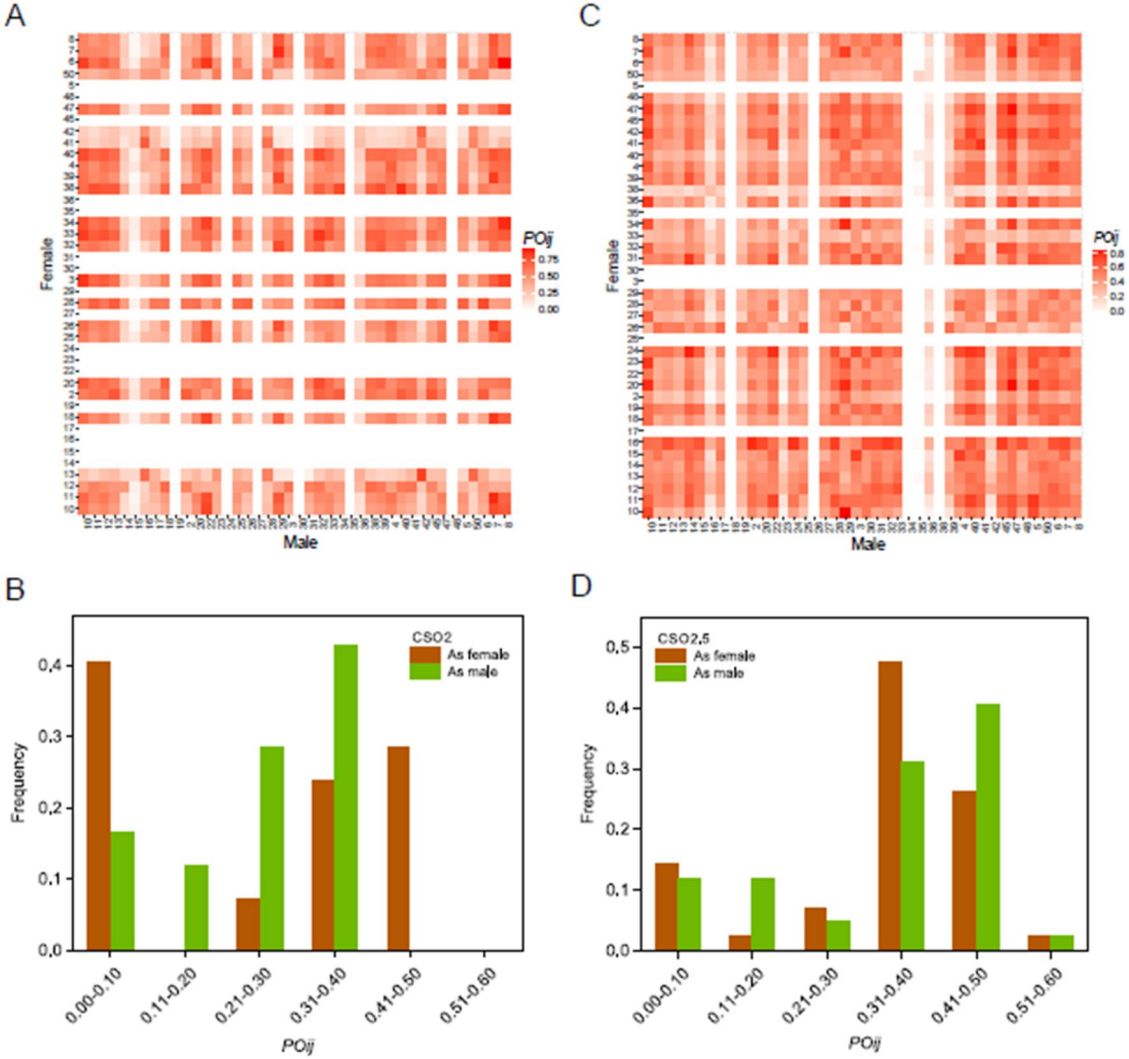
Phenological synchronicity (*POij*) and its distribution of clones for female and male flowering in CSO2 (**A**,**B**) and CSO2.5 (**C**,**D**). The clones listed in (**A**) and (**C**) are ordered by the number of clones.

The average of the synchronization index (*POij*) in CSO2 and CSO2.5 was 0.240 and 0.325, indicating 24.0% and 32.5% overlaps in the time of female receptivity and pollen shedding, respectively (Fig. 4A,C). There were obvious differences in *POij* between males and females in both seed orchards (Fig. 4B,D). The minimum *POij* values for males and females was zero in CSO2 and CSO2.5, indicating that at least one clone of the total had no overlap with any other clones. In CSO2, the mean *POij* value for males was significantly greater than that for females (*F* = 24.07, *P* = 0.0107), indicating that there were more female strobilus available when males were at the peak of pollen shedding than male strobilus available when females were at the peak of receptivity. There was no significant difference in *POij* (*F* = 0.025, *P* = 0.8756) between males and females in CSO2.5. In most clones, there was a similar rank of *POij* between the two seed orchards, such as clone 12 with a high *POij,* as females had a lower *POij* than males in both CSO2 and CSO2.5. The *POij* within clones was higher than the outcrossing combination in both CSO2 (0.337 vs. 0.238) and CSO2.5 (0.357 vs. 0.325), indicating a greater chance of self-pollination. For both female and male strobilus, the *POij* of clones in the early, intermediate, and late classes showed significant differences between the two seed orchards (Table 3). For the male strobilus, the intermediate group had the highest mean value of *POij*.

**Table 3:** Mean phenological synchronization index (*POmean*) values for female and male strobilus in three phenology classes over two seed orchards.

Phenology group	CSO2		CSO2.5	
	*POmean*	SEM	*POmean*	SEM
**Male**				
Early	0.212 cd	0.015	0.322bc	0.024
Intermediate	0.296c	0.008	0.415ab	0.006
Late	0.242 cd	0.037	0.166d	0.007
**Female**				
Early	–	–	0.478a	0.025
Intermediate	0.404ab	0.007	0.318c	0.006
Late	–	–	–	–

“–” means no data, means within generations with different lowercase letters are significantly different at the 0.05 level.

*SEM* the standard error of the mean.

### Correlation and contribution to production

**Figure 5:**
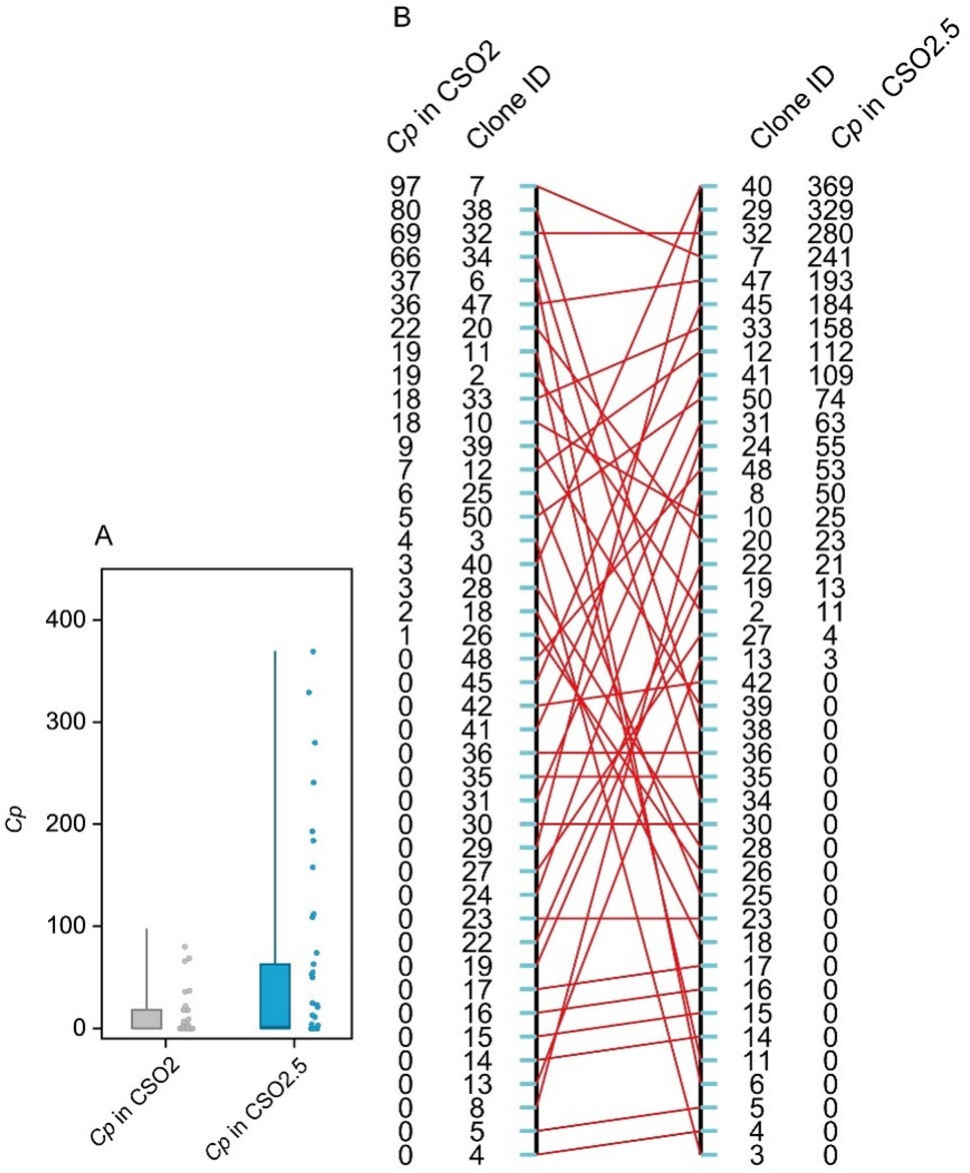
Variation of cone production in two generations seed orchards of Chinese fir. (**A**) The cone production for two seed orchards, (**B**) the rank order for 42 clones based on the cone production in two seed orchards. *Cp* cone production.

**Figure 6:**
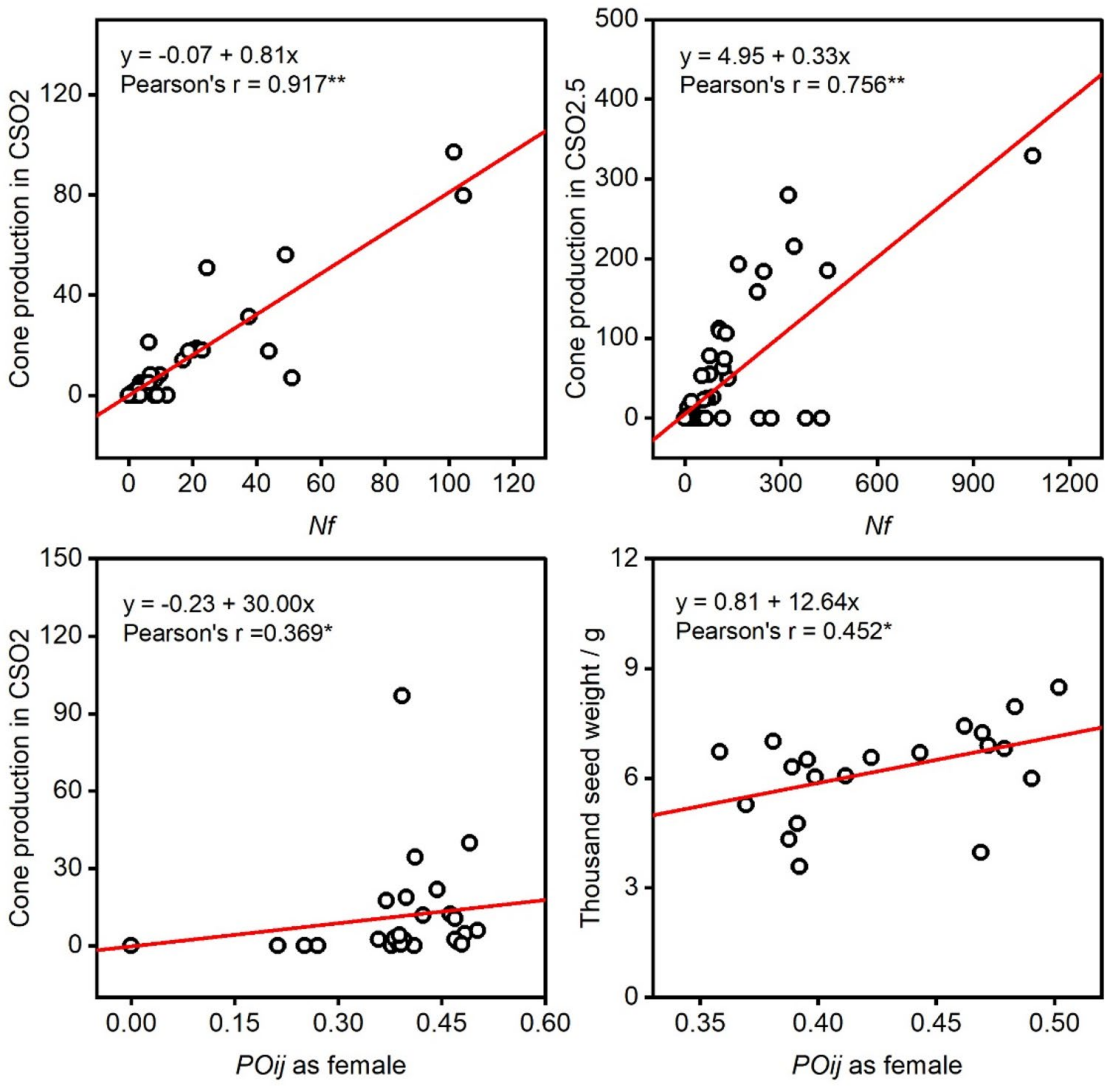
Regression between seed/cone characteristics and number of female strobili (*Nf*), synchronization index in seed orchards. * and ** indicate significant difference at the level of 0.05 and 0.01, respectively.

The cone production (*Cp*) varied between the two seed orchards and among clones within a clonal seed orchard (CSO) (Fig. 5A). A change in cone production rank between the two seed orchards indicated that cone production of clones differed between CSO2 and CSO2.5 (Fig. 5B). There were significant differences in cone production (*Cp*), thousand seed weight (*TSW*), and seed production of one cone (*Psc*) among clones; there were also significant differences in *Cp* and *TSW* between the two seed orchards (Table 4). The high heritability (*H*^2^) of *Psc* indicated that *Psc* exhibited higher genetic control than *Cp* and *TSW* in Chinese fir seed orchards. The results of linear regression analysis showed that there were significant relationships between *Nf* and *Cp* in both seed orchards (Fig. 6). Additionally, there were significant relationships between *POij* as female and both *Cp* and *TSW* in CSO2 but not in CSO2.5 (*r* = 0.120, *P* = 0.447 for *POij* and *Cp*, *r* = − 0.010, *P* = 0.965 for *POij* and *TSW*).

**Table 4:** Analysis of variance for seed production among generations and clones in the seed orchards of Chinese fir.

Source of variation	Variable	*df*	Sum square	Mean square	*F*	*H*_*c*_^*2*^
Generations	*Cp*	1	45,044	45,044	25.77**	0.119
	*TSW*	1	22.116	22.116	21.84**	0.182
	*Psc*	1	0.034	0.034	1.38	0.634
Clones	*Cp*	41	285,386	6961	3.98**	
	*TSW*	30	168.413	5.614	5.54**	
	*Psc*	30	4.77688	0.15923	6.42**	
G*C interaction	*Cp*	41	261,934	6389	3.65**	
	*TSW*	9	28.276	3.142	3.10	
	*Psc*	9	0.5188	0.05764	2.32	
Total	*Cp*	83	592,364			
	*TSW*	40	218.805			
	*Psc*	40	5.330			

*Cp* cone production, *TSW* thousand seed weight, *Psc* seed production of one cone.

## Discussion

### Fertility variation and effective number of parents

Fertility and synchronization are critical in seed orchard design and management and are key to obtaining the potential maximum genetic gains^16^. The number of female strobili (*Nf*) showed significant differences between CSO2 and CSO2.5, and among clones within the seed orchard. The significant age-age correlation of *Nf* between the two generation seed orchards indicated that early prediction was feasible for female strobili in Chinese fir seed orchards. Differences among clones in female and male strobili were also found in other Chinese fir seed orchards, but clones had a positive or negative depending on the years^20^. Fertility can be influenced by genetic and environmental factors such as parent selection, management, tree growth, altitude, and seed orchard age^21^. Tree age and form can affect the number of strobili, pollination, and fruiting by regulating light, nutrient transport, and other factors. In these two seed orchards, difference in tree age and form are the dominant factors inducing the variation in *Nf*. It should be emphasized that tree form control is important for seed production and seed orchard management. The sibling coefficient (*ψ*) value was greater than 2 in two seed orchards, indicating that the probability that more than 2 individuals share a parent is more than twice as high as when the parental fertility is equal across the orchards^4,16,22^. The effective number of parents (*Np*) can estimate the loss of gene diversity between parents and their progeny^1,4^. In CSO2, *ψ* is lower, but *Np* is higher than that in CSO2.5, indicating that seeds from CSO2 would maintain a relatively high level of genetic diversity.

### Phenological synchronization in seed orchards

Synchronization of pollen shedding and female receptivity is one of the major factors affecting genetic gain in seed orchards^2^. On average, pollen shedding and female receptivity occurred over 14 days in both seed orchards, corresponding closely with observations in the Chinese fir clonal archive in Fujian Province, China^23^. In 1994, pollen shedding and female receptivity occurred over 34 days, which was a much longer period than that found in our study^13^. The higher temperature, shorter rainy days, and lower humidity in this study during the flowering period, compared to those in previous study, would induce lower periods of pollen shedding and female receptivity. Female receptivity of clones followed pollen shedding in these two seed orchards, which contributed to crossbreeding in seed orchards as a mean to meet the need for sufficient pollen production. A similar result was also reported for the 1-generation seed orchard of Chinese fir^13^. The significantly positive relationship between male strobili onset and duration of pollen shedding indicated that early blooming clones shed pollen duration longer than late blooming clones. High synchronization is desirable for successful seed production in seed orchards^10^. Our analyses showed that the average value of *POij* was remarkably low but stable across the two seed orchards. Moreover, the synchronization (*POij* = 0.240 in CSO2 and 0.325 in CSO2.5) was obviously lower than that of other coniferous tree species, such as *Pinus patula* (*POij* = 0.49–0.60)^9^, *P. massoniana* (*POij* = 0.513)^24^, and *P. tabuliformis* (*POij* = 0.552)^25^, probably due to the dichogamy and failure of some strobili lowered the *POij* value in our study. In this study, *POij* was mainly distributed from 0.300 to 0.500, implying that there was an imbalance in the gamete contribution and nonrandom cross-fertilization in the seed orchards. Self-pollination is an important factor affecting genetic diversity and genetic gain in seed orchards^2^. In this study, most clones probably had a higher rate of self-pollination, as indicated by the *POij* value in the sample clone being higher than its average *POij* value as a male or female parent. Methods should be considered to dilute this effect when establishing advanced seed orchards, such as deploying clones with similar phenology and increasing the number of ramets per clone. Considered together, pollen contamination seems more important than self-fertilization in affecting the genetic efficiency of the orchard, given the amount of trees releasing pollen in the orchard’s neighborhood^9,12^. Low pollen production and synchronization can create scenarios where some clones are more exposed to contamination by external pollen or less represented in the seed orchard’s progeny^9,26^. In two seed orchards of Chinese fir, the *POij* values were lower than 0.4, which made it possible for clones to be confronted with a high risk of pollen contamination and have a negative effect on genetic gain. The absence of a difference between the *POij* values of two seed orchards indicated the likelihood of pollination due to clone stability from tree growth. This result is similar to the stability in synchronization indices found in Scots pine and radiata pine^11,27^.

### Implication for seed production management

The difference in fertility among parents would cause seed orchard clones to contribute differently to the seed crop^14^. The low value of parental gamete contribution (*U*) (0.204–0.428) indicated an extreme imbalance of female and male gamete contribution. The variation in fertility led to a significant difference in cone production (*Cp*), thousand seed weight (*TSW*), and seed production of one cone (*Psc*) among clones. There were significant positive correlations between *Nf* and *Cp* in both CSO2 and CSO2.5, but no correlations between *Nm* and *Cp*, indicating that the effect of the number and receptivity of female strobili on seed production is higher than male strobili. Therefore, balancing the number of strobili, especially female strobili, should be carefully considered for management in the main production years to obtain high seed yield and quality. For instance, injections or fertilizations of gibberellic acid (GA4/7) can be used to stimulate or enhance strobili production and further enhance cone production. Information about the different clone contributions to the gamete gene pool is important for the proper estimation of the expected genetic composition and genetic gain^11^. The *Cp* and *TSW* were under low genetic control and genetically different, and they varied widely with age and among clones, which should affect the phenotypic diversity of the resulting seeds, as has been shown in other coniferous species^9,17^. These traits may be affected by many factors, such as self-fertilization and synchronization clones^4^. In our case, wide clonal variation in *POij* values as females was significantly associated with *Cp* and *TSW*. The lack of synchronization in the seed orchard provides a warning regarding self-fertilization and genetic contamination risks, particularly for late-flowering clones. These two factors have negative effects on the genetic quality expected in the seeds produced in the seed orchard. However, this negative effect could be reduced by management practices, such as SMP, parent configuration, and hormone application (e.g., GA4/7). Considering that the data were collected in a single year, we should further strengthen the multi-years monitoring of phenology and fertility variation of clones to provide more stability data and improve the genetic gain of seed orchards.

## Methods

### Study site and seed orchards

The study was carried out in two clonal seed orchards (CSOs) of Chinese fir: the 2-generation clonal seed orchard (CSO2) and the 2.5-generation clonal seed orchard (CSO2.5) (Table 5). The CSOs were located in Hongya County, Sichuan Province, China (29.72° E, 103.27° N), and are adjacent to each other. The State Forestry Administration was responsible for national parks and other protected areas. No specific permission was required for these locations/activities, as they utilized non-destructive collection of plant material. The clones in the two seed orchards were identified, bred, and preserved by Sichuan Agricultural University and Hongya State-owned Forest Farm. The species was not endangered or protected, and the locations are not privately owned or protected by law. The collection of plant material complied with institutional, national, and international guidelines. Field studies were conducted in accordance with local legislation.

**Table 5:** Characteristics of the seed orchards.

	2-generation clonal seed orchard (CSO2)	2.5-generation clonal seed orchard (CSO2.5)
Longitude	29.70°	29.73°
Latitude	103.26°	103.27°
Altitude	938 m	978 m
Established	March, 2003	March, 2017
Number of provenance	12	7
Design	Completed randomized blocks	Completed randomized blocks
Repetitions	5	8
Plot	12	15
Espacing	3 × 4 m	3 × 4 m
Mean value of diameter at 1.0 m height	23.78 cm	9.53 cm

The CSO2 was established in 2003 with a completely randomized block design, five repetitions per clone, and a spacing of 3 × 4 m, with 110 elite trees from 12 provenances. The scions were collected from elite trees from 12 provenance locations in Sichuan and Chongqing province, China. They were then grafted on local stock and maintained in Hongya State-owned Forest Farm. The CSO2.5 was established in 2017. The parents were selected from CSO2 according to the results of the progeny test. The CSO2.5 was also established using a completely randomized block design, eight repetitions per clone, and a spacing of 3 × 4 m, with 50 elite parent trees from CSO2.

Forty-two clones (3–4 ramets per clone) were selected and marked for investigation in both CSO2 and CSO2.5. Four standard twigs (the primary lateral twig from the eastern, south, western, and north directions) per tree were marked to investigate the strobili, cone, and seed production and phenology data.

### Flowering and phenology data collection

In 2021, strobili production was investigated by the method described by Chen et al. (1995). The numbers of male (*Nm*) and female strobili (*Nf*) in every tree were surveyed and recorded. The phenological progress of female and male strobili was monitored from March to April 2021. Data were recorded every two days. The phenology of female and male strobili were divided into four stages: the initial stage, peak stage, final stage, and end stage^13^. Male strobili: 1, initial stage (ISm), staminate strobili whitening with little pollen shedding; 2, peak stage (PSm), a large amount of pollen shedding; 3, final stage (FSm), 90% pollen shedding; and 4, end-stage (ESm), 100% pollen shedding and staminate strobili browning. Female strobili: 1, initial stage (ISf), female strobili bud presenting yellow with invisible ovules; 2, peak stage (PSf), at least two wheels of ovuliferous scale expanding and ovule presenting golden yellow with droplet; 3, final stage (FSf), ovule forfeiting fresh with little droplet; and 4, end-stage (ESf), ovuliferous scale completely closing. The trees were divided into three types (early, intermediate, and late type) by the start of pollen shedding and female receptivity^23^. The pollen shedding or receptivity of the early-type tree was two days at the beginning of flowering, the pollen shedding or receptivity of late-type tree referred to the last two days of flowering, all other trees were classified into the intermediate-type group.

### Evaluation of seed production

All cones were picked, and the cone production per entire tree (*Cp*) was calculated. Then, 3–5 cones per tree were randomly selected to measure the seed production of one cone (*Psc*, g). All cones were dried, and seeds were collected to measure the thousand seed weight (*TSW*, g).

### Data analysis

The variance in fertility, phenology, and production were calculated by Genstat 21st edition software through two-way analysis of variance (ANOVA). Within clonal heritability (${H}_{c}^{2}={\sigma }_{c}^{2}/{\sigma }_{total}^{2}$), ${\sigma }_{c}^{2}$ is the estimate of variance for clones, and ${\sigma }_{total}^{2}$ is the total estimate of variance, which includes genetic and environmental variance^10,28^. The correlation between female and male strobili production (*r*) was analyzed by Pearson’s correlation. Afterward, if there was a significant correlation between female and male strobili production then the sibling coefficient (*ψ*), which means combined fertility variation, was calculated by the following formula^29^.

$${\psi }_{f}\,or\,{\psi }_{m} =\frac{{CV}^{2}\times (N-1)}{N}+1$$

$$\psi =0.25\left({\psi }_{f}+{\psi }_{m}\right)+0.5\left[1+r\times \sqrt{({\psi }_{f}-1)({\psi }_{m}-1)}\right]$$

If there was no correlation, the *ψ* value was drawn as follows:

$$\psi =0.25\left({\psi }_{f}+{\psi }_{m}\right)+0.5$$

where ${\psi }_{f}$ expresses the female fertility variation, ${\psi }_{m}$ is the male fertility variation, *CV* is the coefficient of variation of female or male strobili, *N* is the number of female or male parents^22^.

An ANOVA for dichogamy were performed between CSO2 and CSO2.5. The parameters of the effective number of parents in the seed orchard are calculated as follows: the effective number of parents (*N*_*p*_ = *n/ψ*, *n* is the census number of individuals in the seed orchard, the same as below), the effective number of female (*N*_*pf*_ = *n/ψ*_*f*_) and male gametic parents (*N*_*pm*_ = *n/ψ*_*m*_), the relative effective number of parents (*N*_*r*_ = *N*_*p*_*/n*), and the relative effective numbers of female (*N*_*rf*_ = *N*_*pf*_ /*n*) and male parents (*N*_*rm*_ = *N*_*pm*_ /*n*)^4,30,31^. The parental balance curve was used to estimate the uniformity index of female and male gametic contribution (*U*)^32^. The trees were ranked from high to low according to female and male strobili production; then, the cumulative percentage calculations were plotted against the equal cumulative percentage in the seed orchard^5^.

The synchronization index (*POij*) was calculated through the following formula in R^33^.

$${PO}_{ij}=\sum_{k=1}^{n}\mathrm{min}({M}_{ki},{P}_{kj)}/\sum_{k=1}^{n}\mathrm{max}({M}_{ki},{P}_{kj})$$

where *PO*_*ij*_ is the phenological synchronization index between the *i*th and the *j*th clones, *M*_*ki*_ is the proportion of pollen shedding of the *i*th clone at day *k*, *P*_*kj*_ is the ratio of the female receptivity of the *j*th clone at day *k*, and *n* is the days of total strobili duration of *i* and *j*.

Separate ANOVAs for *POij* values were performed at the two seed orchards for female and male strobili. A linear regression analysis between flowering onset and duration was performed to determine the initial stage for phenology duration. A linear regression analysis between strobili production, *PO*_*ij*_, *C*_*p*_, *Psc*, and *TSW* also was performed to determine fertility and phenology synchronization for seed production and quality.

### Ethics approval and consent to participate

All field studies were performed in accordance with the local legislation in China and complied with the convention on trade in endangered species.

## Data Availability

All data generated or analyzed during this study are included in this published article.
